# Clearing up the hazy road from bench to bedside: A framework for integrating the fourth hurdle into translational medicine

**DOI:** 10.1186/1472-6963-8-194

**Published:** 2008-09-24

**Authors:** Wolf H Rogowski, Susanne C Hartz, Jürgen H John

**Affiliations:** 1 Helmholtz Zentrum München, German Research Center for Environmental Health, Institute of Health Economics and Health Care Management, PO Box 1129, D-85758 Neuherberg, Germany

## Abstract

**Background:**

New products evolving from research and development can only be translated to medical practice on a large scale if they are reimbursed by third-party payers. Yet the decision processes regarding reimbursement are highly complex and internationally heterogeneous. This study develops a process-oriented framework for monitoring these so-called fourth hurdle procedures in the context of product development from bench to bedside. The framework is suitable both for new drugs and other medical technologies.

**Methods:**

The study is based on expert interviews and literature searches, as well as an analysis of 47 websites of coverage decision-makers in England, Germany and the USA.

**Results:**

Eight key steps for monitoring fourth hurdle procedures from a company perspective were determined: entering the scope of a healthcare payer; trigger of decision process; assessment; appraisal; setting level of reimbursement; establishing rules for service provision; formal and informal participation; and publication of the decision and supplementary information. Details are given for the English National Institute for Health and Clinical Excellence, the German Federal Joint Committee, Medicare's National and Local Coverage Determinations, and for Blue Cross Blue Shield companies.

**Conclusion:**

Coverage determination decisions for new procedures tend to be less formalized than for novel drugs. The analysis of coverage procedures and requirements shows that the proof of patient benefit is essential. Cost-effectiveness is likely to gain importance in future.

## Background

There is a gap between scientific knowledge and daily medical practice. While there have been major scientific breakthroughs, e.g. in the field of genomics or stem cell research, this does not necessarily directly translate into a variety of new treatments available to patients. A term frequently used to describe approaches for bridging this gap is "translational medicine". Translational medicine faces two major obstacles: the first is the translation of basic science discoveries into clinical studies; the second is to translate clinical studies into medical practice [1]. A large body of literature has focused on the first aspect [2,3]. Yet the second obstacle, which usually depends on coverage by third-party payers, is also essential for the economic success of new products in clinical development.

Coverage determination may prove to be a hurdle as difficult as those encountered in demonstrating the product's efficacy, safety and quality. Due to rapidly increasing healthcare expenditures, numerous countries currently set up institutions that further evaluate new medical technologies after their market approval, before national health services or insurance systems provide coverage [4]. The so-called fourth hurdle set up by third-party payers should thus be considered by those who are involved with earlier stages of medical innovation, e.g. when target conditions and countries are selected and clinical trials are planned.

Looking closely at procedures for coverage determination may demand more attention than a time-constraint clinician or manager can give – the fourth hurdle is highly heterogeneous and subject to frequent reform. Earlier studies have approached the fourth hurdle from a legal or institutional perspective [5,6]. Yet these approaches are quite complex and do not fit easily within the context of the whole process of translation, frequently represented by value-chain steps from basic research to application in healthcare.

This study develops a process-oriented framework to describe fourth hurdle procedures from the perspective of a life sciences company. The framework fits into value-chain representations of technology development and allows for easy presentation and monitoring of these decision procedures. Guidance is derived on how to prepare in advance for the later stages of translational medicine.

## Methods

The study is based on expert interviews, as well as a literature search in PubMed, CRD, Econlit and generally via Google. Relevant attributes of coverage decision-making procedures were extracted and consolidated to a limited number of categories, to fulfil the following, conflicting targets to the highest possible degree:

• Small number, to be manageable

• Conclusive, to describe all items relevant to a life sciences company

• Universal, to facilitate an international comparison of procedures

• Accessible, to enable an investigation of these items for a potential market.

The framework was developed as part of a research project on coverage of novel tissue-engineering (TE) treatments which investigated coverage and reimbursement processes for Autologous Chondrocyte Implantation (ACI) [7-9]. ACI is a novel TE treatment for cartilage defects, particularly in knee joints. Small samples of normal cartilage incorporating cartilage-producing cells (chondrocytes) are removed from the damaged joint, cultured in special laboratories, and re-implanted into the areas of cartilage damage. While ACI may be an effective and cost-effective treatment, the evidence of its long-term benefit is still weak [10]. ACI is the first tissue-engineering treatment that has been subject to explicit coverage decisions in England, Germany and the USA. Most of the current literature is dedicated to the coverage of novel drugs ([e.g. [4,11,12]]), yet innovation in life sciences also encompasses other types of technologies such as procedures applied in hospital. Given the variety of fourth hurdle procedures, the fourth hurdle of these other technologies may not be represented appropriately by procedures aimed at dealing with new pharmaceuticals. The choice of a tissue-engineering procedure as reference case allowed for a framework that is also suitable for other areas of life sciences innovation.

In order to cover various aspects of reimbursement decisions for different types of healthcare funding, institutions in England (tax-based), Germany (social health insurance) and the USA (large share of private health insurance) were chosen. These countries are of outstanding importance to innovative life sciences companies due to their large healthcare expenses. Decisions by the following institutions were selected for comparison: for the English National Health Service, the National Institute for Health and Clinical Excellence (NICE) was selected as the NICE appraisals are relevant for the entire English population and NICE serves as an international reference for fourth hurdle institutions [13]. For Germany, the decisions by the Federal Joint Committee ("Gemeinsamer Bundesausschuss", G-BA) were investigated, which are relevant to approximately 90% of the population who are covered by the statutory health insurance. For the USA, decisions by Medicare were investigated, as Medicare is the largest publicly funded healthcare payer [14] and its decisions strongly influence those of private payers [15]. Private health insurance and out-of-pocket payments account for more than half of the total of USA healthcare expenditures [16]. As an example of a private payer of significant size, the companies of the Blue Cross Blue Shield Association were included in the analysis. 44 websites of different payers in the USA were searched [for details, see: [8,9]].

The selected institutions are highly relevant to life sciences companies due to the healthcare budget within the scope of their decisions. They additionally represent very different approaches to coverage determination. For this reason, it is important to understand the coverage procedures of these institutions before proceeding to a wider international comparison.

Institutions for coverage determination are frequently subject to tight regulation and supervision by government and social courts. This analysis is dedicated to decision-making procedures within the institutions; the legal basis and decisions shaping these institutions were excluded from this analysis. Fourth hurdle procedures are indirectly affected by the rigour of licensing decisions which can vary by type of technology and by jurisdiction. Due to its focus on reimbursement decision making, also the procedures of market approval are beyond the scope of this study.

## Results

**Figure 1:**
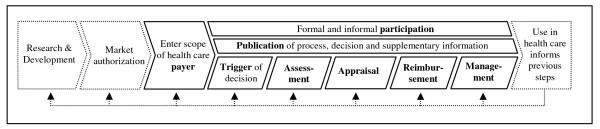
The fourth hurdle within the process of translational medicine.

Reimbursement is both highly heterogeneous across countries and quickly developing at the same time. Figure 1 displays a set of descriptors that serve best to characterize coverage decision-making procedures in international comparison. These descriptors can be seen as crucial steps in the value chain between market authorization and diffusion: (1) after market authorization, a company's product enters the scope of a healthcare payer. (2) The decision maker now has to decide whether, at what cost and under which conditions to cover the technology. As there may be different deciding bodies (e.g. on a national or regional level), the decision process needs to be considered relevant by one specific deciding body before this body's decision process starts. (3) The decision maker typically applies some method of assessing the technology under investigation (e.g. formal reviewing the literature or asking for expert opinion). (4) The information provided by the assessment feeds into an appraisal of whether or not to cover a technology should be covered. (5) After granting coverage, a reimbursement rate needs to be fixed. (6) Besides funding a technology's use, the payer may use other options to exert influence on service provision, e.g. by requiring pre-authorization for expensive technologies. (7) Typically, there are different stakeholders (e.g. doctors or patient groups) to the decision who may be involved formally or informally in the decision process. (8) The decision maker may provide information about the decision process or its outcome during different steps of the decision process. There may be cases where some or even all of these steps are taken simultaneously.

These decisions about coverage, reimbursement and care management will then have a strong impact on the technology's use in health care. Information derived from this use may provide feedback to the different stages of this development: off-label use by doctors may provide information for product development on new target markets [17]; post-approval data may be used for regulatory surveillance [18]; and it may be used to inform or re-start coverage and reimbursement decisions – particularly in the case of coverage with evidence development [19-21].

**Figure 2:**
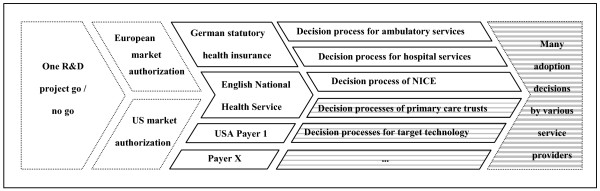
Increasing heterogeneity of decisions in the process of translational medicine.

In the course of the product's life cycle, the decisions involved with proceeding become increasingly heterogeneous: while for many products, market authorization is granted on a supra-national or at least a national level, there are usually several healthcare payers per country. Within the organization of one payer, there can be more than one decision process; e.g. if decisions about new drugs differ from decisions about other technologies or if a formal process is only triggered in case of high budget impact. The heterogeneity further increases over the course of technology diffusion – eventually, decisions of adopting the new technology are made on the level of single healthcare providers or even single doctors. This increasing heterogeneity of decisions is shown in Figure 2.

For an overview of procedures for coverage decision-making in the case of technologies like ACI, see Table 1 below. In the following, further details on the individual steps in the coverage process are provided.

**Table 1:** Overview of the fourth hurdle for ACI

**Payer**	**Germany, Statutory ** **Health Insurance**		**England, National ** **Health Service**	**USA, Medicare**		**USA, Blue Cross ** **Blue Shield**
**Trigger **of coverage decisions	All hospital services are covered unless excluded explicitly. Evaluation upon request of entitled parties (no manufacturers)	All ambulatory services are excluded unless included explicitly. Evaluation upon request of entitled parties (no manufacturers)	Technologies with significant health benefits, social/political effects, impact on NHS resources and added value through guidance by NICE	Initiation of National Coverage Determination: Internally by CMS, external request by interested or aggrieved parties. Only services with considerable impact on the program are evaluated	Initiation of Local Coverage Determination: Internally by the contractor in case of need and in the absence of a NCD	Initiation on company-level, e.g. with Anthem internally by Medical Directors; externally by physicians, manufacturers, HTA organizations
Deciding committee	G-BA, Commission for hospital services [27]	G-BA, Commission for ambulatory services [27]	Independent Appraisal Committee on behalf of NICE [28]	CMS [78]	Contractor's Medical Director [78]	Mostly committee or Medical Director

**Assessment**	Review of evidence of medical benefit in patient-relevant endpoints [15,30,35,42]; HTA potentially by the Institute of Quality and Efficiency in Health Care		Cost-utility model based on all evidence available by contracted technology assessment team; threshold area about £20–30.000/QALY) [79]	Review of evidence of medical benefit; HTA potentially by Agency for Healthcare Research and Quality; by other HTA institution; or by Medicare Coverage Advisory Committee [8]	Review of evidence of medical benefit; HTA potentially by external institution [8]	Review of evidence of medical benefit; HTA potentially by Technology Evaluation Committee or other HTA institution [9]

Criteria for **Appraisal**	Expedience, necessity and efficiency		Effectiveness and cost-effectiveness	Reasonable and necessary	Reasonable and necessary	Medically necessary (Approval, evidence for net health benefits, as beneficial as established alternatives, attainable outside investigational settings)

**Reimbursement**	In-patient: DRGs	Out-patient: Fee for service	Global budgets for PCTs; DRGs paid by PCTs for hospital services	DRGs/Fee-for-service/budgets (MCOs)		

**Management **of service provision	e.g. quality assessment of doctors		e.g. requirement of participation in clinical trial	e.g. preauthorization		

**Participation of company**	Consideration of written comments		Comments about open issues, HTAs and provisional decision; submission of model, appeal possible	Defined periods for comments of all interested parties	Heterogeneous; contractors are required to permit participation	Heterogeneous; mainly participation of medical experts

**Publication**	Amongst others, current open issues, assessment reports and decisions are available through the internet		Process, assessment and appraisal available through the internet	Current open issues and decisions are available through the internet	Written communication in the jurisdiction of the contractor; also available through the internet	Heterogeneous; medical policies often available through the internet

**Abbreviations**: **CMS **Centers for Medicare and Medicaid Services; **DRG **Diagnosis-Related Group; **G-BA **Gemeinsamer Bundesausschuss (Federal Joint Committee); **HTA **Health Technology Assessment; **MCO **Managed Care Organization; **NICE **National Institute of Health and Clinical Excellence; **PCT **Primary Care Trust;

### Payer

Besides out-of-pocket payments, healthcare financing can for example be provided by national and local health services, statutory social insurance systems, private insurance companies, employer-based insurance or integrated service delivery systems with insurance function [22]. The relevance of different types of healthcare payers differs substantially across healthcare systems. Seen from an economic perspective, healthcare payments in England are dominated by one central payer. Also in Germany, the sickness funds within the statutory health insurance system dominate health care financing. For the US healthcare sector, a variety of financing organizations have to be taken into account. To capture the whole relevant market, it is important for a life sciences company to consider all relevant payers for the target healthcare system.

To arrive at an estimate of the relevance of a product's market size covered by the healthcare payer, the number of covered individuals and the types of reimbursed services are of interest to a company. As an example, the German statutory health insurance covers about 90% of the German population. In 2007, its total expenses for health services amounted to £144 billion [23]. While the German statutory health insurance covers individuals at all ages, Medicare in the USA mainly covers the elderly, at a total amount of approximately US$300 billion in 2004. To achieve coverage of technologies primarily for young patients, other payers like the BCBS companies would therefore have to be targeted by a life sciences company.

### Trigger

To be able to tailor its product to the purchaser's preferences, it is relevant for a life sciences company to know who is going to make the decision to finance its product. Coverage decisions are frequently associated with national institutions under the spotlights of politics and the media. Yet due to limited capacities of reviewing bodies, only a small portion of novel technologies is subjected to the resource-consuming formal procedures of national bodies. The vast majority of decisions are usually made at the meso-level of regions or healthcare organizations such as primary care trusts in England or providers' "local coverage determinations" for Medicare in the US. Usually, decisions at the meso-level are far less formalized and thus less suitable for systematic analysis of published documents [24].

Knowing the trigger for a decision-maker's formal evaluation process is, therefore, relevant to determine whether a decision-maker's formal criteria can be expected to be applied at all. Various systems for prioritizing technologies for further investigation can be found across decision makers. Criteria that new technologies have to fulfil to become subject of a formal appraisal process include high-budget impact or disagreement on the technology's effectiveness [25]. In the case of NICE, only technologies with a significant health benefit, impact on other health-related government policies and/or impact on NHS resources are assessed, where the institute is likely to be able to add value by issuing national guidance.

For Germany, primarily the procedure of initiating an appraisal is made explicit: the committee's directives on evaluating technologies only determine the parties authorized to initiate an assessment [26]. Ambulatory treatments are only reimbursed upon explicit acceptance by the Committee – thus, every novel treatment has to pass the committee. The opposite is the case for in-patient services: Here, any novel procedure is covered implicitly within the DRG framework unless it is explicitly excluded by the decision-maker.

Coverage decisions require medical, economic and legal expertise. Thus, they are usually made by interdisciplinary committees. For the German statutory health insurance, decisions are made on behalf of both the Federal Associations of Sickness Fund Physicians or the German Hospital Association and the Federal Associations of Sickness Funds by the G-BA [27]. For the English National Health Service, NICE commissions appraisal committees consisting representatives of relevant stakeholders in the health care system to provide guidance on the technology under investigation [28]. For the decisions on ACI made in the USA, the insurers' medical directors played a major role. Frequently, they rely on the support of an interdisciplinary committee of researchers and other independent experts.

### Assessment

Typically, new technologies undergo some form of assessment of patient benefit or cost-effectiveness prior to the decision. These assessments frequently differ from those used for market approval [29]. In the case of formalized decisions, they often are commissioned out to assessment teams or institutions.

How patient benefit should be established is ambiguous and a topic of persistent controversy, particularly with regard to the use of surrogate endpoints in health-impact measurement and the appropriateness of measurement tools for health-related quality of life [30]. Another point of continual debate is the grading of quality of evidence. Evidence-based medicine advocates the use of high-quality clinical evidence [31]; however, if studies are available, their results tend to reflect a technology's efficacy, while the coverage decision-makers are interested in its "real world" effectiveness in day-to-day medicine. The emphasis of health technology assessments is usually placed on patient-relevant therapy endpoints compared with the current standard of care – as opposed to the frequently used intermediate endpoints in placebo-controlled trials for market approval. Frequently, the full consideration of patient-relevant health outcomes as, for example, changes in residual life expectancy, is beyond the scope of clinical studies. Therefore, decision-makers have to specify their attitudes towards modelling approaches.

Economic evaluations add the issue of value for money and include the costs of a treatment, which implies the assessment of the cost of the respective treatment strategy as a whole, not only the product price. Depending on the type of outcome compared with the costs, different types of studies can be distinguished: cost-benefit analyses measure benefit in terms of willingness to pay; cost-utility analysis use utility values represented by quality-adjusted life years; and cost-effectiveness analyses compare the additional costs associated with a new treatment with the additional health benefit in terms of clinical endpoints [32,33]. A variety of guidelines for economic evaluation have been developed [34]; open issues include, for example, the perspective of the studies or the discount rates for future costs and effects to be applied.

NICE, for example, uses cost-utility models as the state of the art to obtain data for a health technology's effectiveness and cost-effectiveness. Independent academic centres are commissioned to review the published evidence on the technology and synthesize this evidence in decision-analytic models.

The G-BA can commission technology assessments to the Institute for Quality and Efficiency in Health Care (Institut für Qualität und Wirtschaftlichkeit im Gesundheitswesen, IQWiG). This institute also is involved in the development of methods for the evaluation. IQWiG holds a critical attitude towards the use of models and rejected the use of quality-adjusted life years in its statement of methods published in December 2006 [35]. In its statement of "Methods for Assessment of the Relation of Benefits to Costs in the German Statutory Health Care System" published in January 2008, an efficiency frontier approach to economic evaluation was recommended [36]. The methods guidance provides little and partially inconsistent information about the use of decision analytic modelling for evidence synthesis or QALYs for the establishment of effectiveness [37]. Also its revised statement of general methods published in May 2008 does not address the issue of decision-analytic modelling or QALYs and focuses on the establishment of patient benefit on the basis of clinical trials [38].

For companies in the Blue Cross and Blue Shield Association, the Technology Evaluation Center (TEC) is an important source of health technology assessment methods and reports. The use of formal cost-effectiveness analyses has been advocated in the USA [39] and standards for their use in drug coverage decisions have been proposed by the American Academy of Managed Care Pharmacy [40]. Still, their current use in coverage decision-making is rare and assessment of new technologies is mainly based on clinical evidence to establish whether a product improves health outcomes [41].

### Appraisal

These assessments feed into an appraisal of the new technology which may to a large extent be determined by social legislation. For example, according to Book Five of the German Social Code, the G-BA is to appraise whether technologies are "expedient, necessary and efficient" [42]. On behalf of NICE, independent appraisal groups are to appraise the technology's effectiveness and cost-effectiveness; guidance for parts of the additional principles and reflections to account for in the appraisal is provided in a document on social value judgements for NICE appraisals [43-45]. Medicare is to assess whether the technologies are reasonable and necessary; and the BCBS companies are to assess medical necessity [46].

In the case of ACI, the decisions differed across deciding bodies. For example, the G-BA did not reject in-patient treatment with ACI in knee joints so that it is covered by the statutory health insurance [47]. NICE rejected general coverage. However, ACI is being funded as part of a large multi-centre clinical trial to collect further effectiveness data [48]. In the USA, one local coverage determination for Medicare denied coverage of ACI [8]. Among the Blue Cross Blue Shield companies, medical policies regarding ACI differed considerably [9].

### Reimbursement

Market access in terms of coverage may not be sufficient to allow for appropriate funding of a novel healthcare technology. The determination of the level of funding provided by third-party payers is frequently a distinct step, which may involve different decision-making bodies. In some cases (e.g. for new, innovative drugs in Germany), the price requested by the manufacturer is reimbursed directly. Alternatively, insurers may enter into price negotiations with manufacturers as occurs for new drugs in the USA. Besides the amount reimbursed, technical requirements for reimbursement need to be established, e.g. new codes for procedures in fee schedules [49].

Reimbursement can take the form of global budgets, salaries, capitation, case-based payments, fee-for-service or mixed schedules [22]. It has been shown that technology diffusion rates are higher in the case of retrospective fee-for-service reimbursement than for prospective global budgets [50,51].

In a hospital setting, where products like ACI are typically applied, reimbursement is usually in diagnosis-related groups (DRGs) [52]. These groups have been introduced recently in Germany, as well as in the UK. Here, innovations that are cost-saving from the hospital's perspective have a major competitive advantage. For cost-increasing innovation, the application of DRGs to higher expenses may be time-consuming or may fail despite formal coverage of the procedure.

Reimbursement mechanisms can be explicitly designed to accelerate or block the adoption of certain new and more expensive technologies. An example for drug reimbursement provides reimbursement of reference prices instead of manufacturer prices [53]. Examples of mechanisms to accelerate technology adoption in the case of DRG payments are additional reimbursement components for selected technologies [54].

### Management

Reimbursement of a new healthcare technology may come along with specified rules for service provision or quality controls. In general, approaches to connect financing of healthcare to service provision have been termed "managed care" [55]. Examples of tools of managed care are utilization review or standard treatment protocols [22,55]. The stronger this connection, the better the payer can exert influence on the diffusion of new technologies [56,57].

Managed care has played a particularly important role in the USA: most payers offer managed care products. Nevertheless, the number of fully integrated HMO plans has decreased considerably since the end of the nineties [58]; insurance products with traditional fee-for-service payment are available. A tool used frequently by private health insurance in the USA to control service provision is preauthorization, which is especially common with new therapies like tissue-engineering products.

Coverage of new technologies can also be linked with requirements for collecting further data on the technology's effectiveness or cost-effectiveness. This so-called "coverage with evidence development" (CED) balances the aim of offering patients promising new health technologies with the demands for prudent use of resources and the principled arguments that coverage should be based on solid evidence of clinical effectiveness [19,20]. Value of information analysis provides tools to substantiate the decision of whether and which further evidence should be collected to confirm coverage decisions [59]. In the case of ACI, CED has been applied by NICE.

### Participation

Reimbursement decisions are made in the context of a variety of stakeholders' interests [60]. Besides information rights, the major stakeholders may also be involved actively in the decision-making process. The company's formal role in coverage determination procedures may, for example, be the option to initiate a coverage decision, to provide additional information, to participate in public hearings or to appeal within a fixed period.

The decision-making process by NICE is exceptional in terms of stakeholder participation: a manufacturer is offered the status of formal consultee in the appraisal process. This implies that he/she can participate in the "scoping" of the assessment, comment on various documents, submit a report on clinical and cost-effectiveness and appeal against the final appraisal. In decisions by the German G-BA, stakeholder participation beyond doctors and statutory health insurance funds is mainly reduced to comments from medical experts, self-help groups and patient organizations, as well as from umbrella associations of manufacturers.

### Publication

Usually, national bodies publish decision memorandums on websites or in official government documents. In the case of national decisions, published material can go far beyond the decisions themselves. Frequently, decision bodies are additionally obliged to meet standards of procedural transparency and to publish themes of future consultations or documents from ongoing processes. Information on decisions and their rationale is less accessible for decisions at the micro- or meso-level. As far as they are available, these publications provide valuable information for companies both on how to learn best to fulfil requirements for reimbursement and how to keep trace of their competitors.

The English NICE has high standards regarding transparency in decision-making: not only the appraisals and the evidence they are based on, but also a number of documents in the decision-making process are published online. The German G-BA publishes their decisions and the related health technology assessment reports on their website [see e.g. [61]]. The Centers for Medicare and Medicaid services established a database, from which both local and national coverage determinations are accessible .

Table 1 provides an example for a comparative snapshot of the fourth hurdle in Germany, England and the USA. It displays the decision processes including examples of management of services provision for the innovative medical procedure of ACI.

## Discussion

This study presents a simple framework for monitoring fourth hurdle procedures, which consists of eight key steps. Compared with alternative approaches, this framework allows for easy monitoring of these key steps in the process of coverage determination and for easy communication to decision-makers in research and development.

It additionally allows for a comparison of very different processes, e.g. those in English primary care trusts. Even though the majority of coverage decisions are made at this sub-national level, these processes are frequently ignored by the literature.

The framework is best suited to describe formal stages of coverage decisions, based on published documents. Yet usually, health policy decisions are subject to diverse and potentially conflicting political interests [11,24,60,62], and stakeholders like doctors' associations, patient groups or industry representatives may be involved in an informal manner. Frequently, the decisions are, at least to some extent, affected by informal considerations [63] and based on colloquial rather than scientific evidence [64]. Especially for the stages "Trigger", "Appraisal" and "Participation", studies of fourth hurdle procedures can therefore benefit from an inclusion of both formal and informal elements. A substantial part of the variance of health technology decision-making, and thus the probability of success for companies, will remain unexplained by an analysis of overt documents and decision criteria only.

While, on the one hand, the framework is primarily for a descriptive analysis of fourth hurdle procedures, it can also serve as a starting point for normative analysis. Daniels and Sabin propose four criteria for coverage decision procedures in order to meet the standard of "accountability for reasonableness" [65]: (1) Decisions regarding coverage for new technologies and their rationales must be publicly accessible (publicity condition); (2) These rationales must rest on evidence, reasons, and principles that all fair-minded parties can agree are relevant to deciding how to meet the diverse needs of a covered population under necessary resource constraints (relevance condition); (3) There must be a mechanism for challenge and dispute resolution regarding limit-setting decisions (appeals condition); and (4) There is regulation of the process to ensure that the first three conditions are met (enforcement condition). Applying the framework presented in this study to coverage decision processes additionally delivers information on their accountability for reasonableness, because it includes descriptive information for criteria (1)–(3) as proposed by Daniels and Sabin.

### Relation of process and outcome

The coverage decisions for the reference case study are heterogeneous: Coverage is provided by the German statutory health insurance for hospital care and by a number of private payers in the USA if certain criteria are met. Coverage was declined by NICE (yet the development of further evidence is supported), as well as by the one Medicare local coverage determination.

One major reason for this heterogeneity may be the ambiguous evidence both for the effectiveness and cost-effectiveness of ACI: there are studies that suggest ACI is effective, but the long-term effectiveness and cost-effectiveness remain to be established [10]. The differences may also be explained partly by the payers' scope: as a major potential benefit of ACI is presumed to be sustainability as compared with implants, it is of higher relevance for younger patients than for individuals over the age of 65. Therefore, Medicare would be more likely to decline coverage, than would benefit schemes for younger patients.

Interestingly, the German G-BA arrived at different conclusions for different conditions: while coverage for ACI in finger and shoulder joints was denied, the coverage decision for ACI in knee joints was postponed until 2014, with no conclusive evidence of long-term effectiveness for either condition. The explanatory statement justified this decision according to anticipated new evidence, particularly from studies in England. This can be taken as an example of the substantial amount of unexplained variance in coverage decisions, yet may also partially be explained by cooperation among coverage decision-makers; apparently, the G-BA is waiting for the results of further clinical studies conducted on behalf of NICE.

### How to pave the way from bench to bedside

To pave the way for a research project from bench to bedside, project managers should prepare for the stages through which a novel technology will have to pass before being applied in healthcare. This framework provides background information and can help identifying important issues to address. These include, for example, which decision procedure is likely to be triggered by the product. If formal decisions on a macro-level are likely, clinical trials should be designed to include the endpoints and potentially the treatment alternatives requested by the relevant fourth hurdle processes.

The level of detail in which these questions can be answered will depend on the stage of project development [66]. Early in research, it may be sufficient to do an explorative search of HTA reports for potential target conditions. This provides a first overview of potential medical need, practice patterns and competitors. Closer to the clinical trial stage, it may be worthwhile to develop early health technology assessments. A number of analytical tools are available for purposes like early market assessment, R&D portfolio management and first estimations of pricing and reimbursement scenarios. Nevertheless, high uncertainty of clinical and economic data in early stages restricts the validity of modelling results [67,68]. As soon as sufficient clinical data are available, formal health economic models can be used to establish information needs for further clinical trials or scenarios for cost-effective pricing strategies.

### Limitations of this analysis

Any overview of coverage decisions can only be a snap-shot, as institutions and requirements for coverage of new medical technologies are subject to frequent, considerable changes. This is illustrated by recent reforms in all countries included in this review, e.g. the introduction of formal cost-effectiveness analysis for novel drugs in Germany which was passed in parliament in February 2007. In June 2008, the methods of cost-effectiveness analysis were still subject to debate [36]. Therefore, the results presented in this article's case study, as well as for any company's target health technologies, warrant regular updating.

The US healthcare system is highly heterogeneous and hosts a multitude of payers, many of which have different processes and requirements. Even though the case study presented above investigated major players, findings can hardly be generalized.

Besides the processes described in Table 1 of this study, further decision processes exist – in the case of Germany for example for ambulatory drug reimbursement or dental care; in England for example the procedures within primary care trusts. Even if the framework is generic, the overview in Table 1 had to focus on those processes which made decisions about ACI. It therefore does not provide a conclusive overview but an example of fourth hurdle processes.

The EMEA and the FDA have different practices for licensing [69,70], and for other technologies like medical products other procedures and criteria for market approval exist [71]. More rigorous licensing practices may filter out technologies that otherwise have to be assessed by fourth hurdle institutions. Therefore, these procedures which are not in the scope of this study have an influence on procedures for coverage and reimbursement.

In general, the procedures involved in the translation of clinical research from bench to bedside are internationally very heterogeneous and complex. Therefore, any simple framework can be claimed to lead to over-simplification and there are research questions for which the framework is not the best choice. For a detailed institutional analysis of coverage decision-makers, the framework developed by Hutton and colleagues would be better recommended because it can provide more precise answers [6]. The framework also has its limitations if rather than one specific technology, an overview of coverage of very different types of technologies, e.g. within one country, is to be provided. Within this process-oriented framework, these decisions would all be described as a multitude of single decision processes. This might jeopardize the framework's simplicity. It is, therefore, better suited for a technology-specific description of the fourth hurdle – which corresponds with the need of a project manager to have the technology life cycle of a single project in mind, rather than a country's system for coverage determination. To describe entitlements to health benefits for a country, an approach like the one chosen by the Health Basket Project may be more appropriate [42].

The diffusion of health technology is a dynamic process between patients, physicians, hospitals, healthcare systems and technology. Apart from a product's effectiveness or cost-effectiveness, important impact factors in this process include purchasing power in the target country [50] or commercial marketing efforts [72,73]. Some of these impact factors will also appear in an application of this framework, e.g. as informal decision criteria (e.g. affordability) or informal participation (e.g. influence by the industry). Nevertheless, researchers interested in that area should draw upon models and approaches developed in the literature on health technology diffusion [50,72-74].

## Conclusion

### Implications for researchers and life sciences companies

All over the world coverage decision-makers are developing procedures to prioritize healthcare spending. Project managers involved in the translation of research projects from bench to bedside should keep track of the relevant fourth hurdle procedures in their target countries as part of their marketing activities. The framework presented above provides a tool to describe and compare these procedures in a transparent and manageable manner.

The focus of a decision-maker's interest is the evidence-based establishment of a technology's effectiveness in terms of patient-relevant endpoints. Of the three countries under investigation in the case study, the use of formal cost-effectiveness analysis was to a large extent restricted to NICE in England – yet due to increasing need for prioritization as a result of escalating healthcare costs, the high international perception of NICE and the evolving use of formal cost-effectiveness analysis for drug treatments [4,11,39,75], it can be assumed that its importance will increase in future for other healthcare technologies.

Life sciences companies should include final health outcomes in their clinical trials for market approval, to provide information on patient benefit in order to achieve coverage. They should additionally collect resource consumption data, so that they can provide health economic evidence if required.

### Implications for policy-makers

As shown in Figure 2, project managers are confronted with a variety of heterogeneous fourth hurdle procedures that lead to different outcomes. To enhance translational medicine, it would be desirable to increase the consistency of fourth hurdle procedures across payers and healthcare systems.

It has been reported that the criteria for establishment of effectiveness and value for money are not always applied consistently [15,76]: decisions may be inconsistent with stated requirements, as for ACI-coverage in Germany, or they may be inconsistent between different deciding bodies, as for ACI coverage by Blue Cross Blue Shield companies in the USA. It is important that efforts to standardize fourth hurdle procedures do enforce, rather than jeopardize, their accountability for reasonableness [77], and lead to the adoption of those health services that are most desired by patients and the population.

To enhance the translation of research findings, it would be desirable if institutions for research funding would increase awareness of later stages in the project development process. This could be conducted, for example, if as a part of advertisements for research opportunities, information on targeted markets and potential reimbursement strategies was requested.

### Implications for further research

There are a variety of open research questions on the different process steps. The framework presented here can assist researchers in identifying and distinguishing different areas of research at the important intersection of translational medicine and health services research. Two major areas of further research on the whole process of translation should be addressed: first, fourth hurdle institutions have been subject to continual reform. Thus, the framework presented above should be applied on a regular basis to keep track of changing conditions of market entry and diffusion. The framework was applied only to one technology and a limited number of decision-makers in three countries. It would, therefore, be of additional interest to include further decision-makers and reference technologies in future analysis. Second, coverage is a crucial target for the marketing activities of life sciences companies. It would be of value to develop further guidance on how requirements for coverage can be integrated into innovation management in a more systematic manner. Given the escalating healthcare costs in industrialized countries and the rising importance of fourth hurdle procedures to prioritize healthcare resources [4], it can be expected that the need for tools and guidance is likely to grow in the future.

## Competing interests

This research was carried out on behalf of the Helmholtz Zentrum München, German Research Center for Environmental Health (HMGU) and was partly funded by the German Federal Ministry of Education and Research. The HMGU is an independent organization funded by the German and Bavarian government. During the time of this study, all authors were employees of the HMGU and neither did nor do not have a conflict of interest with regard to this project.

## Authors' contributions

WR conducted the searches for Germany and the UK, developed the framework and wrote the manuscript. SH conducted the searches on the fourth hurdle in the USA and helped to draft the manuscript. JJ was involved in all stages of the project and provided substantial input on the framework, the sources and validity of the data used and the conception of the manuscript. All authors read and approved the final manuscript.

## Pre-publication history

The pre-publication history for this paper can be accessed here:


